# NGF and NGF-receptor expression of cultured immortalized human corneal endothelial cells

**Published:** 2010-07-29

**Authors:** Federica Sornelli, Alessandro Lambiase, Flavio Mantelli, Luigi Aloe

**Affiliations:** 1 Institute of Neurobiology and Molecular Medicine, Department of Neurobiology, National Research Council (CNR), Rome, Italy; 2 Department of Ophthalmology, University Campus Bio-Medico, Rome, Italy

## Abstract

**Purpose:**

Several growth factors, including nerve growth factor (NGF) and vascular endothelial growth factor (VEGF), play an important role in the homeostasis of the ocular surface. The involvement of both these growth factors in the pathophysiology of intraocular tissues has been extensively investigated. Despite the expression of NGF receptors by corneal endothelium, to date the role of NGF on the endothelial cell remains to be determined. Using a clonal cell line of human corneal endothelial cells, the aim of this study was to investigate the expression of the NGF-receptor and the potential partnership of NGF and VEGF in maintaining cell viability in vitro.

**Methods:**

A human endothelial cell line (B4G12), was cultured under serum-free conditions as previously described with and without addition of different concentrations of NGF, anti-NGF-antibody (ANA), or VEGF for 4 days and these cells were used for immuno-istochemical, biochemical, and molecular analyses.

**Results:**

NGF induces overexpression of NGF-receptors and synthesis and release of VEGF by endothelial cells and these cells are able to produce and secrete NGF.

**Conclusions:**

These observations indicate that human corneal endothelial cells are receptive to the action of NGF and that these cells may regulate NGF activity through autocrine/paracrine mechanisms.

## Introduction

Degeneration of corneal endothelial cells is a critical pathogenetic event of a wide number of ocular surface diseases, from congenital, to inflammatory, immune and degenerative. The result of an altered corneal endothelium function is, inevitably, a progressive loss of corneal transparency leading to blindness. Therefore, once the total count of endothelial cells is not sufficient to warrant corneal transparency, surgical intervention with a corneal transplant is currently the only option available, since corneal endothelial cells do not have the ability to proliferate. Several growth factors present in the anterior chamber of the eye have been investigated for their potential role in supporting endothelium survival and function. Nerve growth factor (NGF) is the first discovered and best-characterized member of the neurotrophin family [1]. It is produced by and acts upon cells of the visual system, both in vitro and in vivo and it is able to promote the functional recovery of retinal ganglion cells (RGCs) in an animal model of ocular ischemia and following optic nerve section, to reduce retinal cell damage induced by intraocular hypertension and to delay retinal cell degeneration in rodents with retinitis pigmentosa [2-7]. These effects are mediated by two NGF-receptors, the high-affinity receptor tyrosine kinase (TrkA), and the low-affinity receptor p75 neurotrophin receptor  (p75), both located on the surface of NGF-responsive cells. Altered expression of these receptors and/or their ligands can lead to NGF-target cell degeneration [8]. NGF is present in the aqueous humor, increases following ocular injuries, and binds to its specific receptors expressed by the corneal endothelium. It has also been demonstrated that topical NGF eye drops administration promotes corneal healing and exerts anti-inflammatory and immunomodulatory actions on corneal endothelial cells [9-11]. Another growth factor that has been extensively investigated in the last years for its effects in modulating ocular immune and healing processes is the vascular endothelial growth factor (VEGF). VEGF is an endogenous biologic mediator that is released by endothelial cells and is known to play a pivotal role on ocular disorders and corneal vascularization [12-18]. Recent studies have shown that NGF, like VEGF, possesses angiogenic and neurotrophic action and is able to activate an intracellular signaling cascade in endothelial cells, the Ras/extracellular signal-regulated kinase (Ras/ERK) and phosphatidylinositol 3-kinase-dependent (P13/Akt) pathways, involved in the survival and in the modulation of angiogenic activity [19,20]. Moreover, previous studies have also indicated that VEGF plays a role in mediating corneal nerve repair and the detrimental effects of anti-VEGF drugs on the ocular surface are mediated by a down regulation in NGF levels [21,22]. These observations and recent evidence that *NGF* gene transfer to the corneal endothelium modulates endothelium survival through the inhibition of immune reactions triggered us to investigate the physiologic role of NGF on corneal endothelium survival both directly through binding to its receptors, and/or indirectly through VEGF [11]. The aim of the present study was, therefore, to investigate the effect of NGF in an in vitro human corneal endothelial cell line that displays several characteristics of in vivo human endothelial cells [23].

## Methods

### Chemicals

NGF, anti-mouse NGF-antibody and VEGF (Sigma-Aldrich, St. Louis, MO) were used for cell treatment. Purified NGF was isolated from mouse submandibular gland following the method of Bocchini and Angeletti [24]. The anti-mouse NGF antibody was prepared in rabbits and purified by affinity chromatography and characterized as described in another study [25]. Polyclonal rabbit anti-trkA (1mg/ml; diluted 1:50; Up State, Temecula, CA), monoclononal mouse anti-VEGF (1mg/ml; diluted 1:50; Santa Cruz Biotechnology, CA), monoclonal mouse anti-p75 (clone 192; diluted 1:10) purified in our laboratory [26] and biotinylated goat anti-rabbit or horse anti-mouse IgG (Vectastain Elite ABC Kit; Vector Laboratories, Inc. Burlingame, CA) using the procedure suggested by the manufacturer were used for immunocytochemistry, while the goat anti-rabbit 594 or anti-mouse 488 IgG antibody Alexa Fluor (diluted 1:250; Invitrogen, Eugene, OR) and Hoechst 33258 (5 μg/ml; Sigma-Aldrich, St. Louis, MO) were used for immunofluorescence.

### Cell line and cell culture

A clonal cell line (B4G12) of human corneal endothelial cells (HCEC), indicated thereafter as HCEC, was kindly obtained by Dr. Monica Valtink, from the Institute of Anatomy, Medical Faculty, Technishe Universitat Dresden, Germany. Cells were cultured in complete medium in T25 culture flasks coated with 1 mg chondroitin-6-sulfate (from shark cartilage; Sigma-Aldrich) and 10 μg laminin (Sigma-Aldrich) as suggested [27]. At confluence, cells were removed using trypsin/EDTA (0.05%/0.02%) and enzyme activity quenched by proteinase inhibitor cocktail [23]. For our studies, cells were cultured on T25 culture flasks pre-coated as previously described in serum-free medium Human Endothelial-SFM **(**GIBCO-Invitrogen Carlsbad, CA) supplemented with 10 ng/ml human recombinant basic fibroblast growth factor (bFGF) (Sigma-Aldrich). Cells were seeded at a density of ~8,000 cells/cm^2^ with or without NGF, anti-NGF antibody (ANA), or VEGF at different concentration and counted 4 days later. Cells were removed with Trypsin/EDTA solution (Sigma-Aldrich) at 37 °C and 5% CO_2_ for 3 min and its activity quenched by trypsin inhibitor (Sigma-Aldrich). Cell viability was evaluated using Trypan blue exclusion (16–910–49; Flow laboratories Irvine, Scotland, UK), a vital stain. Only cells which excluded trypan blue were counted.

For immunolocalization, cells were cultured on chamber slides (NUNC, Rochester, NY). The cells in culture were observed under a Leica DM IRB microscope (Leica Microsystems, Bannockburn, IL) and analyzed with SPOT Advanced program.

### NGF and VEGF determination

Cell pellets obtained by centrifugation of cell cultures (150× G for 5 min) were homogenized in extraction buffer (10 mM tris-HCl, pH7.4, 100 mM sodium-chloride, 1 mM ethylenediamine-tetraacetic acid [EDTA], 1 mM ethyleneglycol-tetraacetic acid [EGTA], 1% triton X-100, 10% glycerol, 0.1% sodium-dodecil-phosphate, 2 mM sodium-ortovanadate, 20 mM sodium-pyrophosphate, 1 mM sodium-fluoride, 2 μg/ml aprotinin, 1 mM phenylmethylsulfonyl fluoride [PMSF], 1 µg/ml leupeptin), and supernatant was clarified by centrifugation at 4 °C at 8,000× G for 20 min. The cell concentration of NGF and VEGF was measured using an ELISA kit (DuoSet Human β-NGF and Human VEGF kit; R&D Systems, Minneapolis, MN) following the instructions provided by the manufacturer. Data are represented as pg protein/µg of protein and all assays were performed in triplicate.

### Single immunocytochemistry

Cultured cells were washed with phosphate buffer saline (PBS), fixed in 4% paraformaldehid (PFA) for 10 min, washed twice in PBS, and then permeabilized with PBS containing 0,1% Triton X-100 for 30 min. Cells were then exposed to 10% methanol containing 3% H_2_O_2_ in PBS for 15 min followed by exposure to BSA (BSA 1%) in PBS for 10 min, and incubated overnight at 4 °C with the following primary antibodies: polyclonal anti-TrkA, monoclonal anti-VEGF, monoclonal anti-p75, anti-NGF antibody, all at a concentration of 2 μg/ml. After brief washes in PBS, cells were incubated with specific biotynilated secondary antibody and then with immunoperoxidase with the ABC Vectastain Kit (Vector Laboratories, Inc. Burlingame, CA) as suggested by the manufacturer’s instructions. Staining was developed by exposure to a diaminobenzydine H_2_0_2_ mixture for 15 min and covered with mounting medium. Staining specificity was assessed by omission of the primary antibody and by isotypic IgG. Cell immunoreactivity was evaluated under a 40× objective frame, with a Zeiss Axiophot microscope (Carl Zeiss S.p.A., Milano, Italy) connected to a PC and an image analysis program (IAS 2000; Delta Sistemi, Rome, Italy). The number of VEGF, TrkA, and p75 positivity cells in different fields (n=12) and different experimental groups (n=4) was counted and evaluated. The results are expressed as a percentage of positive control cells.

The effect of NGF on VEGF expression was investigated after immunoflorescence staining and HCEC were observed with confocal microscopy (Leica SP 5;  Leica Microsystems, Wetzlar, Germany).

### Double immunocytochemistry

To assess the existence of co-expression of VEGF, TrkA, and p75, fixed cultured cells were first immunostained as above with VEGF, then immunostained with TrkA or p75 following the same procedure. The presence of the second antibody was revealed with a different biotynilated probe. Cells were finally washed, mounted with glycerol 50% in PBS (E. Merck, Darmastad, Germany) on a glass and visualized under microscope equipped with 40× objective. All stained cells were then examined with a Zeiss Axhiphot microscope and images photographed and acquired with an image analysis program (IAS 2000; Delta Sistemi, Rome, Italy).

### Western blotting

Cell pellets obtained by centrifugation of cell cultures (150× g for 5 min) were homogenized in extraction buffer (10 mM tris-HCl, pH7.4, 100 mM sodium-chloride, 1 mM ethylenediamine-tetraacetic acid [EDTA], 1 mM ethyleneglycol-tetraacetic acid [EGTA], 1% triton X-100, 10% glycerol, 0.1% sodium-dodecil-phosphate, 2 mM sodium-ortovanadate, 20 mM sodium-pyrophosphate, 1 mM sodium-fluoride, 2 μg/ml aprotinin, 1 mM phenylmethylsulfonyl fluoride [PMSF], 1µg/ml leupeptin), and supernatant was clarified by centrifugation at 4 °C at 8,000× g for 20 min and used for western blotting. Samples (30 µg of total protein) were dissolved with loading buffer (0.1 M Tris-HCl buffer pH 6.8 containing 0.2 M DTT, 4% SDS, 20% glycerol and 0.1% bromophenol blue), separated by 8% SDS–PAGE and electrophoretically transferred to polyvinylidene fluoride (PVDF) membrane overnight. The membranes were incubated for 1 h at room temperature with blocking solution (5% BSA in TTBS 10 mM Tris, pH 7.5, 100 mM NaCl, and 0.1% Tween-20: 10 mM Tris pH 7.5, 100 mM NaCl and 0,1% Tween-20: 10 mM Tris, pH 7.5, 100 mM NaCl, and 0.1% Tween-20: 10 mM Tris, pH 7.5, 100 mM NaCl, and 0.1% Tween-20). Membranes were washed three times for 10 min each time at room temperature in TTBS followed by incubation at 4 °C with primary antibody overnight. Membranes were washed three times for 10 min in TTBS and incubated for 1 h a room temperature with secondary antibody anti-rabbit IgG HRP linked.

The blots were developed with ECL chemiluminescent HRP substrate (Millipore Corporation, Billerica, Ma) as the chromophore. A personal computer and the public domain image J software were used to evaluate band density, which was expressed as arbitrary units of gray level. The image J software determines the optical density of the bands using a gray scale thresholding operation. The optical density of glyceraldehyde 3-phosphate dehydrogenase (GAPDH) bands was used as a normalizing factor. For each gel/blot the normalized values were then expressed as a percentage of the relative normalized controls and used for statistical evaluation.

### Real time RT–PCR

HCEC treated and untreated with NGF or ANA were removed with Trypsin/EDTA solution at 37 °C and 5% CO_2_ for 3 min and its activity quenched by trypsin inhibitor. Then the cells were centrifuged at 150× g for 5 min to obtain a pellet which was then stored a −80 °C until further use. Total RNA from cells was extracted using a kit, SV Total RNA Isolation System (Promega Italia, Milan, Italy), following the instructions provided by the manufacturer. RNA was quantified by spectrophotometry at 260 and 280 nm and the samples stored at −80 °C. RNA was converted into cDNA in a 25 μl reverse transcription reaction containing 1 μg of total RNA, 1× reverse transcriptase buffer (50 mM Tris-HCl pH 8.3, 75 mM KCl, 3 mM MgCl_2_, 10 mM DTT), 0.5 mM dNTPs, 500 ng oligo (dT)_15_, 40 U Rnasin RNase inhibitor, and 200 U of M-MLV RT. Reactions were incubated at 42 °C for 60 min, heated at 95 °C for 5 min, then cooled at 4 °C for a minimum of 5 min and a maximum of 30 min and the sample stored at −20 °C. RT–PCR was performed using the 7900HT Fast Real-Time PCR System (Applied Biosystems, Branchburg, NJ), FAM-labeled probe specific for the human TrkA (Assay ID Hs00176787_m1), FAM-labeled probe for human Glyceraldehyde-3-phosphate dehydrogenase (*GAPDH*) (Assay ID Hs99999905_m1) as endogenous control and TaqMan Universal PCR Master Mix (Applied Biosystem) was included in the reactions.

The cDNA was amplified under the following conditions: 1 cycle at 50 °C for 2 min and at 95 °C for 10 min, followed by 40 cycles at 95 °C for 15 s and at 60 °C for 1 min. Negative controls (without template) were produced for each run. Experiments were performed in duplicate for each data point. The amount of mRNA of each gene was calculated using the standard curve method (following the instructions in User Bulletin no. 2; Applied Biosystems) and adjusted for the expression of *GAPDH*.

### Statistical analysis

Data were obtained by means of ANOVA (ANOVA) using the Stat View package for Macintosh (Abacus Concepts Inc., Berkeley, CA) considering the resting condition of NGF and VEGF, the effects of NGF, ANA, and VEGF on HCEC survival at different culture conditions, the effects of NGF and ANA on VEGF and TrkA expression, as variables. Differences between groups were determined by the posthoc Tukey-Kramer test. All results are reported as a mean±standard error of the mean (S.E.M.). A p-value less than 0.05 was considered significant.

## Results

### HCECs express NGF receptors

**Figure 1:**
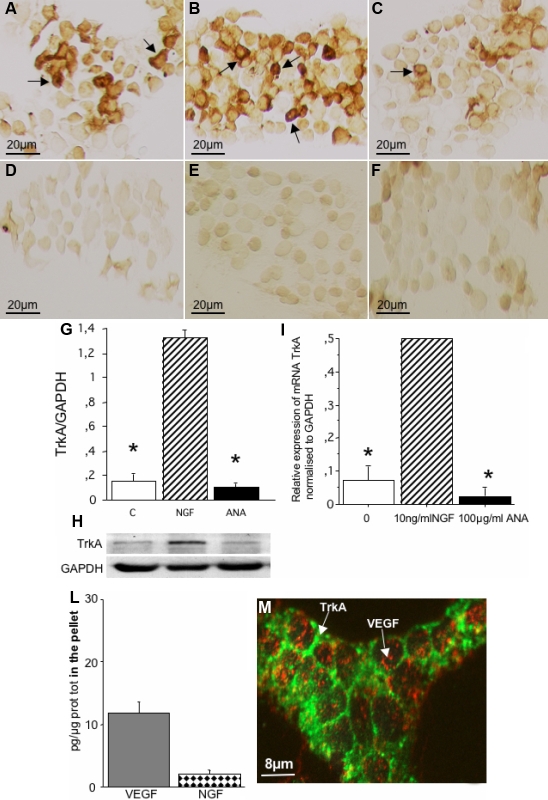
Representative immnocytochemical preparations showing the expression of NGF-receptors in HCECs cultured in vitro for 4 days under different conditions. TrkA expression in control condition (**A**), TrkA expression in cells exposed to NGF (**B**) and TrkA expression in cells exposed to ANA (**C**). Note that NGF enhances and ANA down-regulates the expression of TrkA. Panel **D** illustrate the expression of the NGF-receptor p75 in control conditions, in cells exposed to NGF (**E**), and in cells exposed to ANA (**F**). This NGF-receptor is nearly non-expressed in control HCECs (**D**), it is unaffected after treatment with NGF (**E**) or ANA (**F**). Scale bars: **A**-**F** 20 μm. Panels **G** and **H** report the results of western blot and real-time PCR of HCECs treated with NGF or ANA. As indicated in **G** and **H**, compared to control, HCECs exposed to NGF express more, while these exposed to ANA express less TrkA protein. The PCR analysis also revealed that NGF enhances and ANA reduces the expression of TrkA gene expression (**I**). Panel **L** reports the amount of NGF and VEGF expressed by HCECs under cultured for 4 days without NGF. This result indicates that these cells constitutively express, though differently, both NGF and VEGF. As illustrated in **M**, confocal immunohistochemical analysis revealed that HCECs express VEGF (red) and TrkA (green). Scale bar: **M**=8 μm.

We first investigated whether HCEC express the NGF-receptors TrkA and p75. As reported in Figure 1A-F, HCECs express the high-affinity NGF receptor, TrkA, in resting condition (Figure 1A), TrkA increases after exposure to NGF (Figure 1B) and decreases after exposure to ANA (Figure 1C). The low-affinity NGF receptor, p75, is poorly expressed both in basal condition (Figure 1D), and after exposure to NGF (Figure 1E) and ANA (Figure 1F). Western blot analysis and real time PCR also indicated that NGF increases and ANA decreases TrkA-protein expression (Figure 1G,H) and TrkA gene expression (Figure 1I).

### HCECs produce VEGF and NGF and co-express VEGF and TrkA

As shown in Figure 1L, immunoenzymatic determination of cell pellet cultured for 4 days without NGF indicated that these cells express 12.4±0.8 pg/mg of VEGF protein and 4.2±0.3 pg/mg of NGF protein.

Confocal immunohistochemical analysis revealed that HCECs express VEGF (red) and TrkA (green) Figure 1M.

### NGF promotes HCEC survival in a dose-dependent manner

**Figure 2:**
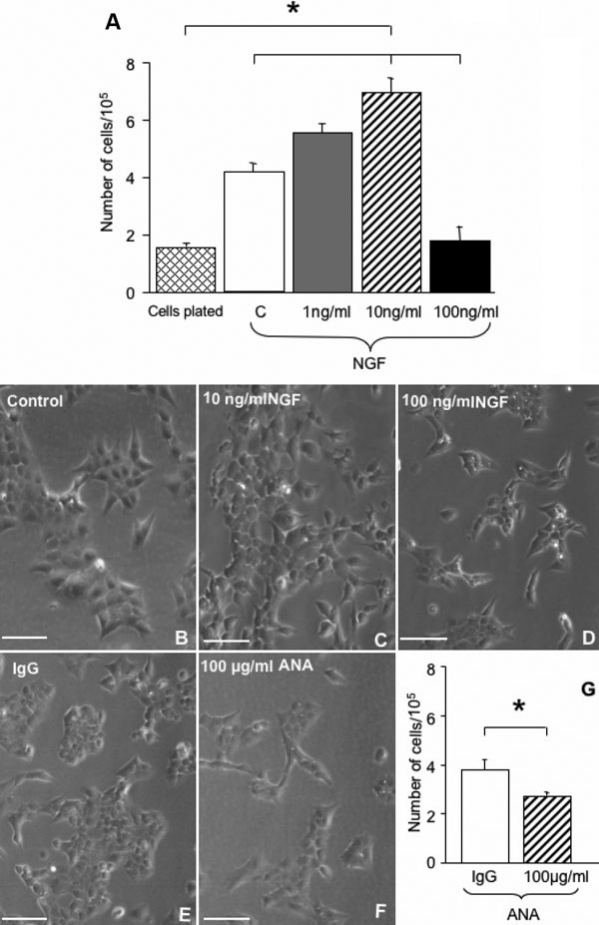
This figure reports the effect of NGF on HCEC survival. **A**: The result of a time-course analysis of HCECs exposed for 4 days to culture medium alone or with 1 ng, 10 ng, or 100 ng of NGF/ml, revealed that at 1 and 10 ng, NGF enhances cell survival, while 100 ng/ml of NGF down-regulates cell survival, compared to control, (*p<0.05). **B**-**D** illustrate the structural aspect of these cells exposed to medium alone (**B**), 10 ng/ml (**C**), and 100 ng/ml of NGF (**D**). Note the decreased in cell number in **D**. Panels **E** and **F** illustrate the effect of endogenous NGF inhibition by ANA on HCEC survival. The supplementation of ANA in the culture medium at concentration of 100 ng/ml of medium, significantly (*p<0.05), reduces the number of cells after 4 days in vitro (**F**), compared to control (**E**). This effect was statistically significant (**G**). Scale bars: **B**-**F**=25μm.

A dose–response analysis indicated that NGF exposure for 4 days promotes HCEC survival in a dose-dependent manner. As reported in Figure 2A, the presence of 1 and 10 ng/ml of NGF stimulates cell survival, while 100 ng/ml of NGF has no stimulatory action. This latter effect is not due to HCEC death, but to inhibition of cells proliferation (data not shown). The morphological characteristics of HCECs cultured in vitro in the presence or absence of NGF is shown in Figure 2B-D.

To further investigate the role of NGF on HCEC survival, these cells were exposed for 4 days to 100 µg/ml ANA, or pre-immune serum (IgG). As reported in Figure 2E-G, the presence of ANA reduces the number of HCECs (Figure 2F), compared to cells exposed to IgG (Figure 2E). This effect is statistically significant, as seen in Figure 2G.

### NGF stimulates the release of VEGF

**Figure 3:**
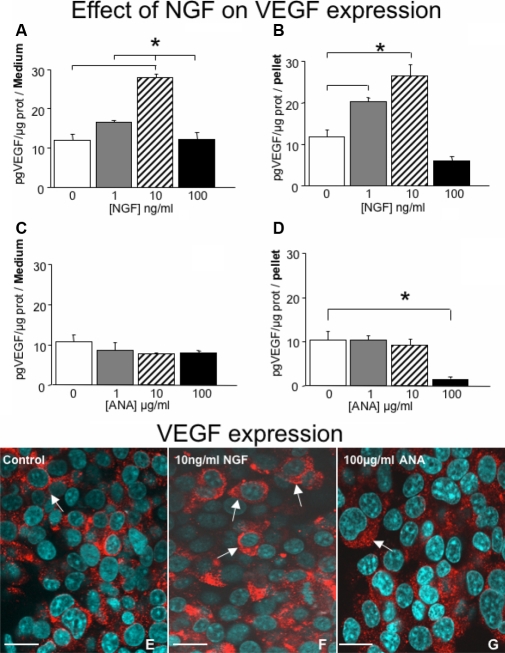
Dose–response effect of NGF and ANA on VEGF expression in culture medium and cell pellet of HCECs after supplementation of 1, 10, and 100 ng/ml of NGF or ANA at 1, 10 and 100 μg/ml of medium. NGF enhances the presence of VEGF both in the medium and in pellet at concentration of 1 or 10 ng/ml of NGF. At 100 ng/ml NGF has an inhibitor action on VEGF expression. ANA exposure has no effect on the concentration of VEGF in the medium (**C**), but significantly reduces (*p<0.05) VEGF presence in the pellet at concentration of 100 ug/ml (**D**). Confocal microscopic analysis of cells exposed to 10 ng/ml of NGF and 100 µg/ml of ANA are illustrated, respectively in **F** and **G**. Note the enhanced expression of VEGF after exposure of NGF (**F**) and the down-regulation after exposure of ANA (**G**), compared to control (**E**). Arrows point to VEGF immunopositivity. Scale bars: **E**-**G**=15μm.

We next tested the effect of NGF on cultured HCECs on VEGF expression. As reported in Figure 3, NGF stimulates the release of VEGF into the medium (Figure 3A) and in cells (Figure 3B), in a dose-dependent manner. The effect of VEGF stimulation, both in cells and medium, is more conspicuous at the concentration of NGF 10 ng/ml and nearly absent at 100 ng/ml. Thus, like the effect on cell survival, NGF has no action also on VEGF stimulation at high concentration NGF has no action, most probably due to saturation of NGF-receptors expressed by HCECs. To further investigate this effect HCECs were cultured for 4 days in presence of different concentration of ANA. The results shown in Figure 3C,D, indicated that ANA has no effect on VEGF release into the medium at all concentrations tested (Figure 3C), while it exerts a significant down-regulation of VEGF expression in cell pellet at the concentration of 100 ng/ml (Figure 3D). This latter effect might be due to the inhibition of the biologic activity of NGF and NGF-receptors constitutively present in HCECs.

Confocal microscope analysis of HCECs also revealed that NGF at 10 ng/ml enhances the expression of VEGF in the cells, Figure 3F, while 100 μg/ml of ANA reduces the expression of VEGF, Figure 3G, compared to untreated cells, Figure 3E.

### The effect of NGF on HCEC survival is different from the effect of VEGF

**Figure 4:**
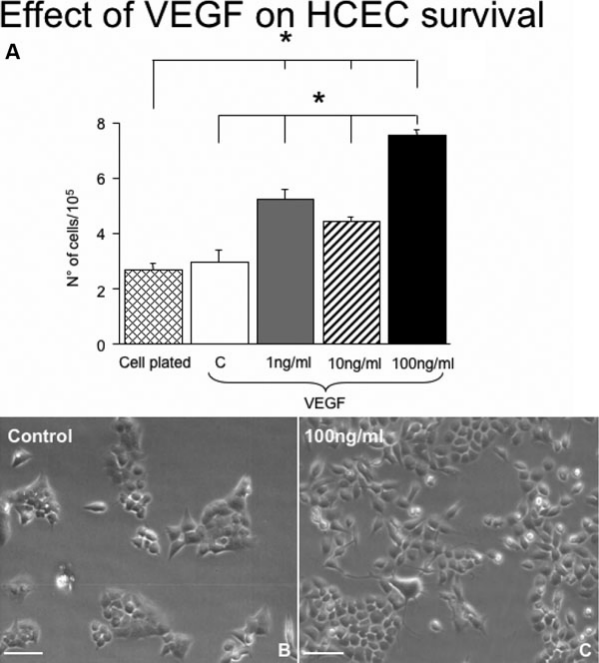
Dose–response analysis on the effect of VEGF on HCEC survival. The results indicated that VEGF supplementation into culture medium for 4 days enhances HCEC survival at 1, 10, and 100 ng/ml medium, compared to control (**A**; *p<0.05). The morphological aspect of HCECs exposed to control medium without and with 100 ng/ml of VEGF is shown in **B** and **C**, respectively. Scale bars: **B**, **C**=25μm.

Because NGF and VEGF seem to have similar effects on endothelial cell survival, we next tested the effect of VEGF on HCEC survival at 1, 10 and 100 ng/ml of medium [16,19]. As reported in Figure 4, a dose–response study indicated that VEGF enhances the survival of HCEC in a dose-dependent manner (Figure 4A), exerting its highest effect at the dose of 100 ng/ml of medium. Differently from NGF, VEGF has no inhibitory action at high concentration. The survival and morphological aspect of HCECs cultured in presence of medium only and 100 ng/ml of VEGF of medium are illustrated in Figure 4B,C.

## Discussion

In this study we investigated the role and the effects of NGF on a human corneal endothelial cell culture. Our results indicate that the human corneal endothelial cell line B4G12 produces and releases NGF and that NGF enhances the expression of VEGF and the high-affinity NGF-receptor, TrkA, but not the low-affinity NGF-receptor, p75. It is well known that the TrkA/p75 ratio regulates cell fate and function, with p75 being pro-apoptotic and TrkA promoting cell survival in different cell types [8,28]. Our evidence of NGF promoting TrkA –but not p75– expression in HCEC may explain the observation that NGF promotes HCEC cell survival in a dose-dependent manner, while the neutralizing NGF antibody has the opposite effect. The expression of NGF and both its receptors by the same cell line suggests that these effects are regulated by an autocrine or paracrime mechanism.

It is plausible to hypothesize that these beneficial effects physiologically occur following intra-ocular injuries (as previously demonstrated in experimental iridectomy) to protect the corneal endothelium, in line with previously published data on NGF endothelial gene transfer and NGF effects during corneal epithelial injury and wound healing [11,29].

The effects of NGF on corneal endothelial cell survival may also be synergistic with those of other growth factors physiologically present and/or released following intra-ocular injuries. Among these growth factors, in this study we investigated the role of VEGF since increasing evidence is demonstrating a direct partnership of NGF and VEGF in the regulation of cell function, healing and immune mechanisms.

VEGF has been primarily known as an angiogenesis mediator, however increasing evidence is now also showing that VEGF might play relevant roles in cell survival. In line with these recent updates on VEGF function, our in vitro data demonstrates a survival-promoting effects of VEGF on HCECs. It may also be interesting to speculate that this survival-promoting effect of VEGF could be amplified in vivo by an autocrine/paracrine NGF stimulation of VEGF production and release in the anterior chamber of the eye.

Based on this data, another fascinating hypothesis that can be drawn involves an active role of both NGF and VEGF on the pathophysiological mechanisms behind the immune reactions of the anterior chamber of the eye. In fact, it is well known that the anterior chamber represents a site of immune privilege (the so-called ACAID or Anterior Chamber Associated Immune Deviation) in which, to date, the endothelium has not been considered to play an active immunomodulatory action.

If confirmed, this presumed endothelial-driven immunomodulation could be related to our evidence on VEGF in HCECs, since VEGF has been recently demonstrated to play immunomodulatory roles, including the maturation of granulocyte-macrophage progenitors and on the inhibition of dendritic cells’ development into mature, antigen presenting, dendritic cells [30]. In addition, NGF has also been shown to play an important role in regulating the immune response. Specifically, NGF modulates the immune reaction form predominantly Th1 to Th2 type and exerts a relevant anti-inflammatory action.

The fact that both VEGF and NGF are expressed by corneal endothelial cells supports the hypothesis that these growth factors are implicated in the physiopathology of corneal homeostasis. Moreover, the observation that ANA reduces the number of HCECs and down-regulates the expression of TrkA and VEGF, further supports the hypothesis that NGF plays a critical action on corneal homeostasis and healing, most likely acting not only on corneal epithelial cells –as already widely demonstrated by our group and by others– but also on endothelial cells.

Why ANA is unable to alter the basal expression of TrkA and VEGF is not known. One possibility is that other mechanisms are involved in the regulation of TrkA and VEGF expression by corneal endothelial cells.

In conclusion, the results of this study provide additional evidence that human corneal endothelial cells are NGF target cells that express both NGF receptors. An autocrine/paracrine mechanism of NGF on these receptors may trigger not only the well known effects of NGF on corneal healing and survival, but also initiate more complex signaling pathways involving other growth factors (such as VEGF) with immunomodulatory roles.

These observations might facilitate the development of further pharmacological approaches of NGF-based therapy and possibly improve the prognosis of corneal endothelial diseases and the outcomes of corneal transplantation. Taken together our findings suggest that NGF eye drop administration, might contribute not only to epithelial cells proliferation, but also to the maintenance of corneal endothelial function [10,31].
